# Lutein and Zeaxanthin and Their Roles in Age-Related Macular Degeneration—Neurodegenerative Disease

**DOI:** 10.3390/nu14040827

**Published:** 2022-02-16

**Authors:** Małgorzata Mrowicka, Jerzy Mrowicki, Ewa Kucharska, Ireneusz Majsterek

**Affiliations:** 1 Department of Clinical Chemistry and Biochemistry, Medical University of Lodz, 90-419 Lodz, Poland; malgorzata.mrowicka@umed.lodz.pl (M.M.); jerzy.mrowicki@umed.lodz.pl (J.M.); 2 Department of Gerontology, Geriatrics and Social Work, Jesuit University Ignatianum, 31-501 Krakow, Poland; ewa.kucharska@ignatianum.edu.pl

**Keywords:** lutein, zeaxanthin, xanthophylls, carotenoid deficiency, age-related macular degeneration, oxidative stress

## Abstract

Lutein and zeaxanthin belong to the xanthophyll family of carotenoids, which are pigments produced by plants. Structurally, they are very similar, differing only slightly in the arrangement of atoms. Key sources of these carotenoids include kale, savoy cabbage, spinach, broccoli, peas, parsley, corn, and egg yolks. The recommended daily intake of lutein is approximately 10.0 mg and that of zeaxanthin is 2 mg. Lutein intake in adults varies, with average intakes being 1–2 mg/day. Due to the lack of synthesis of consumption of these compounds in humans, these substances are extremely important for the proper functioning of certain organs of the body (eye, skin, heart, intestines). Eating a lot of dark leafy vegetables and some fruits can help to prevent our bodies from developing diseases. The protective effects of carotenoids are mainly related to their defense against oxidative stress and their ability to scavenge free radicals. Lutein and zeaxanthin are the only dietary carotenoids that accumulate in the retina, specifically the macula, and are called macular pigments. These carotenoids are concentrated by the action of specific binding proteins such as StARD3, which binds lutein, and GSTP1, which binds zeaxanthin and its dietary metabolite, mesozeaxanthin. It has been shown that supportive therapy with lutein and zeaxanthin can have a beneficial effect in delaying the progression of eye diseases such as age-related macular degeneration (AMD) and cataracts. This article presents the current state of knowledge on the role of lutein and zeaxanthin, especially from human studies targeting their metabolism and bioavailability, with recommendations to consume xanthophyll-rich foods.

## 1. Introduction

Carotenoids can be divided into carotenes, e.g., α-carotene, β-carotene, lycopene, torulene, isorenieratene, and their oxygen derivatives, xanthophylls, containing oxygen in the molecule in the form of hydroxyl, epoxy, or carbonyl groups, e.g., lutein, zeaxanthin, canthaxanthin, astaxanthin, and echinaxanthin. Due to the presence of hydroxyl groups in the carbon ring in the molecule, compared to carotenoids, xanthophylls are more polar compounds, absorbing radiation of shorter wavelengths. Xanthophylls are widely distributed in nature, with a group of compounds showing both chemical and physicochemical similarity. The presence of a minimum of seven double bonds in the chain determines that carotenoids are yellow to red coloring substances in both plants and animals. Carotenoids are insoluble in water, but are very soluble in fats or non-polar solvents, with which they often form esters.

Unlike plants, animals cannot synthesize them independently through biochemical processes.

Chemically, lutein is an unsaturated polyene hydrocarbon composed of eight isoprene residues, forming a carbon chain with 40 carbon atoms and two—OH groups in β-ionone rings. The carbon skeleton has two ends, and both have a molecule containing a cyclic hexenyl structure with an attached hydroxyl group. The structure of lutein, with its nine double bonds, allows it to absorb certain wavelengths of light and emit other wavelengths, leading to the characteristic color properties of these molecules. The main isomer of lutein found in fruits and vegetables is its *trans* form, but significant isomer changes occur during processing [1].

**Figure 1:**
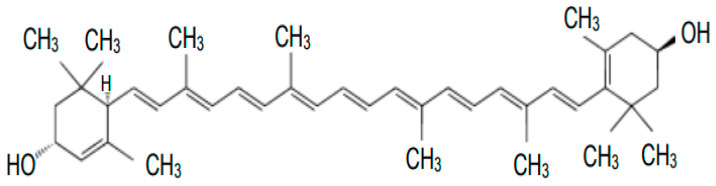
Structural formula of lutein.

The sum formula of lutein is C_40_H_54_O_2_. Figure 1 shows the structural formula of lutein.

**Figure 2:**
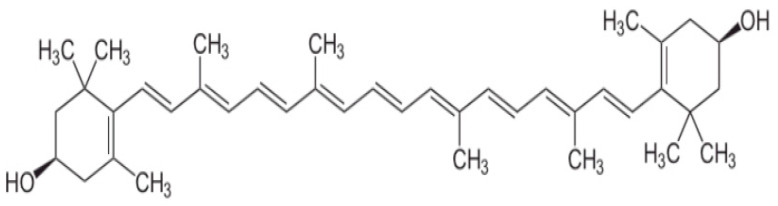
Structural formula of zeaxanthin.

Zeaxanthin is an isomer of lutein that differs in the location of the double bond in the cyclic ring. Figure 2 shows the structural formula of zeaxanthin.

Lutein and zeaxanthin can only be synthesized by plants—green vegetables, especially kale, spinach, lettuce, and broccoli, are high in their content (Table 1). Therefore, these substances must be supplied to the body with the diet. The content of lutein/zeaxanthin in vegetables varies greatly and ranges from 0.01 to 40 mg/100 g. The highest content is found in kale (~39 mg/100 g) and spinach (~11.9 mg/100 g). In addition, lutein/zeaxanthin is found in lettuce, broccoli, Brussels sprouts, parsley, zucchini, peppers, and egg yolk. Vegetables are a better source of these xanthophylls than fruits. Nectarines, blackberries, avocados, raspberries, gooseberries, kiwi fruits, and black currants have the highest content among fruits [2,3].

To date, the daily requirement for lutein has not been established. Neither a Lowest Observed Adverse Effect Level (LOAEL) nor a No Observed Adverse Effect Level (NOAEL) has been determined, as no clinical studies have demonstrated side effects associated with high doses of lutein in humans. According to EFSA experts, the safe intake of the synthetic form of zeaxanthin is 0.75 mg/kg body weight per day, which corresponds to a daily intake of 53 mg for a person weighing 70 kg [4].

## 2. Metabolism and Functions of Carotenoids

More than 600 types of carotenoids occur in nature, 10% of which are part of the normal human diet. However, only a small proportion of these are routinely detected in human serum. Carotenoids are synthesized in living organisms by phytoene synthase, which produces the 40-carbon core of carotenoid molecules, carotene desaturase, which forms double bonds between adjacent carbons, and carotene cyclase, which catalyzes the reaction to attach six-carbon rings to the ends of the 40-carbon backbone [5].

Most carotenoids, 95% of them, are synthesized by the condensation process of two molecules of digeranyl diphosphate (GGPP, C20PP), resulting in a symmetric C40 phytoene backbone. This reaction is catalyzed by the enzyme phytoene synthase. Then, in the presence of oxidases, phytoene undergoes a four-step conversion into a lycopene molecule. The cyclization reaction produces α-carotene and β-carotene. In subsequent processes, β-carotene is converted to zeaxanthin by a hydroxylation reaction, and α-carotene is converted to lutein. Enzymes required for carotenoid production are encoded in the genomes of many bacteria, unicellular eukaryotes, fungi, and plants. Additional enzymes found in groups of organisms can lead to modifications, such as the attachment of a hydroxyl group or the shortening or lengthening of the carbon skeleton [5]. Animals require the presence of carotenoids as provitamins for proper vision processes and skin coloration due to the carotenoids themselves. However, most of them do not possess genes encoding enzymes necessary for carotenoid biosynthesis, so they supply these compounds from food. Factors affecting the bioavailability, absorption, transport, storage, and catabolism of carotenoids primarily include the type, the number of carotenoids, and the environment in which they are absorbed. The bioavailability of carotenoids is determined by genetic factors, sex, age, health and nutritional status, or the release of these compounds from food [6].

Lutein (free or esterified) and zeaxanthin are fat-soluble, and fat is needed both for their more efficient absorption in the small intestine and their transport via the bloodstream to various body tissues. Before being absorbed by enterocytes, lutein esters are hydrolyzed by gastrointestinal enzymes such as cholesterol esterase. Intestinal absorption of carotenoids, which are highly hydrophobic molecules, involves steps similar to those of fatty acids and vitamins soluble in them. Heat treatment of plant foods destroys cell membranes and protein complexes of carotenoids and increases the bioavailability of carotenoids. These compounds are relatively resistant to elevated temperatures, so heat treatment of foods does not reduce their biological properties. In addition, the bioavailability of lutein and zeaxanthin is facilitated by finely chopping foods and cooking foods [1]. The uptake of non-esterified lutein by enterocytes is thought to occur through a process involving the scavenger receptor class B type 1 (SR-B1) [7]. Carotenoids are encapsulated in the inner space of mixed micelles to enhance their solubility, absorption by the intestinal mucosa, incorporation into chylomicrons, and release into the lymph. After carotenoids are released from chylomicrons by lipoprotein lipase, they are transported by high-density lipoproteins (HDL), low-density lipoproteins (LDL), and, to a lesser extent, very low-density lipoproteins (VLDL). Lutein is mainly stored in adipose tissue and hepatocytes. It is assumed that lutein is excreted through the bile and kidney [8].

Approximately 20 different types of carotenoids have been identified in human plasma or serum. In the diet and blood, non-polar carotenoids (called carotenes) make up most of the carotenoid pool. As a result of selection and accumulation, only two are found in the retina, lutein and zeaxanthin, which are not found in high concentrations in the blood or in the average diet. The concentration of these xanthophylls in the human retina and lens is many times higher than in other tissues [9]. Lutein and zeaxanthin are the only carotenoids reported to be present in the eye lens. The range of ratios of lutein to zeaxanthin in the human normal lens is 1.6 to 2.2. This proportion of lutein and zeaxanthin is similar to the human retina ratio of lutein and zeaxanthin [10].

However, relatively high amounts have also been reported in the adrenal glands, corpus callosum, lungs, testes, and skin. Furthermore, Vishwanathan et al. (2016) [11] showed that lutein is the predominant carotenoid in brain tissues and its action is associated with cognitive function in adults. The authors also showed that lutein and mesozeaxanthin concentrations in the occipital cortex are dependent on their concentrations in the macula.

Carotenoids, through the presence of a conjugated double bond system, exhibit strong antioxidant activity, act as free radical scavengers, and are involved in photoprotection. The ability to remove harmful singlet oxygen is proportional to the number of conjugated double bonds in the carotenoid molecule [10]. It has also been shown that lutein and zeaxanthin are found in the skin and subcutaneous tissue in a hybridized form and protect against UV and reactive oxygen species.

Macular xanthophylls are mainly localized in retinal membranes around Henle’s fibers, the layer formed by photoreceptor axons, and in the membranes of photoreceptor outer segments. The localization of carotenoids in the prereceptor layer is attributed to the role of a filter against harmful short-wave blue light. Their presence in receptor membranes (rich in polyunsaturated fatty acids and prone to peroxidation) is associated with their antioxidant function. Carotenoids as membrane antioxidants are very good singlet oxygen quenchers and could neutralize free radicals [10]. The structural and dynamic properties of the biomembrane are more susceptible to oxidative degradation by ROS, which can be reduced by the direct action of carotenoid pigments.

Moreover, the structure and high chemical reactivity of carotenoids make them capable of exerting bioprotective effects on the human body. These chemical compounds are involved in many beneficial processes, such as stimulation of the immune system, modulation of intercellular signaling pathways, regulation of the cell cycle, apoptosis, regulation of growth factors, and helping in the fight against viruses [12,13,14,15]. Xanthophylls stimulate intercellular communication through nexus-like connections—protein channels in cell membranes—modulate signaling pathways, and stabilize cell membranes by binding lutein and zeaxanthin to microtubules in the cytoskeleton [16,17]. These compounds affect the immune system, increasing the number of defense cells, T cells, and cytotoxic T cells. Lutein has been shown to decrease the number of defense cells, T cells, and cytotoxic T cells. Lutein has been shown to reduce cancer risk or inhibit cancer cell growth [18].

## 3. The Role of Lutein and Zeaxanthin in Eye Vision

Two tissues of the eye that play a key role in the vision process are the macula and the lens. Of all the carotenoids present in the human diet and serum, only two, lutein and zeaxanthin, are present in these two important tissues. In 1945, George Wald analyzed the macular pigment (MP) and discovered that lutein, zeaxanthin, and its oxygen form, mesozeaxanthin, were responsible for the yellow color. The distribution of lutein and zeaxanthin in the retina of the eye varies. Zeaxanthin is the dominant carotenoid in its central part, and lutein in its peripheral parts.

Zeaxanthin is the dominant macular xanthophyll in the center of the fovea, an area with a high density of cone photoreceptors that is exposed to bright light [19,20]. Li et al. [19] documented that the distribution of lutein is more diffuse throughout the macula, at a reduced concentration, compared to the level of zeaxanthin in the fovea. Just as lutein and zeaxanthin are exogenous components, mesozeaxanthin is not derived from the diet. It is likely that mesozeaxanthin is formed from lutein in the retina. Lutein undergoes enzymatic or photochemical conversion to mesozeaxanthin. Mesozeaxanthin can be synthetically produced from lutein by the enzyme RPE65 (retinal pigment epithelium-specific protein) in the RPE [21]. This metabolite is not found in human plasma or liver but is present in ocular tissues, suggesting specific metabolic pathways in the eye [22,23]. Studies in monkeys also confirm the conversion of lutein to mesozeaxanthin in the retina [24].

The macula lutea, or “macula,” due to its yellow color, is in the central and posterior parts of the retina. The highest concentration of macular pigment is observed near the fovea because zeaxanthin and mesozeaxanthin are the dominant macular xanthophylls in the center of the fovea. Lutein is the main xanthophyll of the peripheral part of the retina. Zeaxanthin concentrations are 2.5-fold higher than lutein in the cone-dense central fovea [20].

Each of the two carotenoids is retinally uptaken by separate binding proteins. The GSTP1 isoform has been identified as a local retinal zeaxanthin-binding protein. Lutein is found in many tissues, but the relative amount of this xanthophyll in the retina is the highest among tissues. A lutein-binding protein was recently isolated from the human peripheral retina and shown to interact with antibodies against a protein belonging to the steroidogenic acute regulatory domain (StARD) family. Finally, StARD3 (out of 15 known human StARD proteins) was found to be a human retinal lutein-binding protein [20,25,26]. The third xanthophyll-binding protein is tubulin. Tubulins are abundantly present in the axons of photoreceptor cells (Henle fibers) and this is where macular pigment is mainly localized in the human retina.

**Figure 3:**
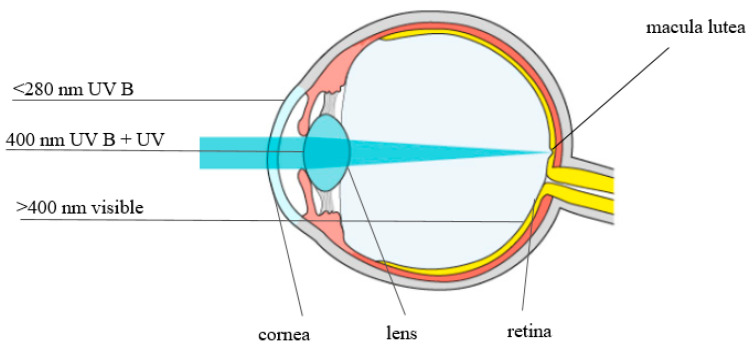
Absorption and transmission of solar radiation in the eye.

Many researchers have argued that lutein and zeaxanthin protect the retina and lens from age-related changes [25,27,28]. Most UV radiation is absorbed by the lens; wavelengths between 400 and 700 nm (visible light) and 700 and 1400 nm (infrared radiation) are readily transmitted by the human ocular structures to the retina (Figure 3).

Maintaining macular health is critical to maintaining normal visual function. Light-induced retinal damage depends largely on wavelength, exposure time, and power level, with 440 nm blue light requiring 100 times less energy to cause damage than 590 nm orange light [29]. There is an inverse relationship between macular pigment density and lens density in the eye, suggesting that macular pigment may serve as a marker for xanthophylls in the lens. Lutein, zeaxanthin, and mesozeaxanthin are absorbers of blue visible light (400–500 nm) and thus protect the eye structures from dangerous doses of this radiation. The ability to absorb visible blue light is due to the structure of these compounds. This optical filtration is particularly important because short-wavelength (blue) light is highly reactive and could enhance photooxidative degeneration in the most sensitive neurosensory layers of the retina [30].

The effect of macular pigments on the retina is to protect it from oxidative processes when exposed to light. This property has been demonstrated in studies on rats, quail, frogs, and monkeys [22,31,32].

In addition, a very important property of macular pigments is their ability to inhibit aberration and improve the contrast sensitivity of the objects we look at. Contrast sensitivity (CS) is the ability to discriminate visual stimuli based on their frequency. The eye fails to discriminate between stimuli when their frequency is too high or too low. Carotenoids (L and Z) are thought to pre-filter blue light to reduce the adverse effects of glare disorders, light scattering, and chromatic aberration, thus optimizing contrast sensitivity. Macular pigments act as filters in that they improve vision by increasing both the contrast of objects and their background [33,34,35]. Stringham et al. [33] reported that supplementation with a macular carotenoid product increased contrast sensitivity, improving glare incapacity. L and Z also increase luminance and reduce the effect of reflected luminance. The reduction in glare correlates significantly with macular pigment concentration [36,37].

## 4. Lutein, Zeaxanthin, and Macular Degeneration

Age-related macular degeneration (AMD) is the degradation of the central part of the retina, including the macula, and is the leading cause of blindness in older people in developed countries. AMD can be divided into two categories: early (or dry AMD) and late (or wet AMD). AMD can manifest as geographic atrophy (GA) in the dry form or choroidal neovascularization (nAMD) in the wet form. The disease begins as a dry form and evolves to a wet form in 10% to 20% of affected individuals.

In 2010, an estimated 2 million patients in the United States had AMD. This number is expected to increase to 3.6 million and 5.4 million in 2030 and 2050, respectively [38]. A study by Colijn J.M. and co-authors [39] found a trend toward a slightly decreasing prevalence of AMD in older adults in Europe. Using meta-analyzed data from 14 population-based cohort studies included in the European Eye Epidemiology (E3) consortium, the authors showed improvements in visual acuity in nAMD occurring over the past two decades in Europe after 2006. Projections for AMD show an almost doubling of the number of people affected, despite the decreasing prevalence. By 2040, the number of people in Europe with early AMD will range from 15 to 21.5 million and with late AMD from 3.9 to 4.8 million.

Globally, 196 million patients and 288 million patients are expected to have any form of AMD in 2020 and 2040, respectively [40].

The predominant risk factors for developing AMD are age, sunlight exposure, smoking, and poor nutritional status [41,42,43]. Late AMD often leads to visual disability in the form of blindness, and there is currently no effective therapy.

AMD is a multifactorial disease of the photoreceptor support system, which includes the retinal pigment epithelium (RPE), Bruch’s membrane, and choroidal vessels. Age-related macular degeneration is a disease in which immune and complement pathways and lipid transport, extracellular matrix remodeling, angiogenesis, and impaired DNA repair, apoptosis, and oxidative stress are abnormal.

The amount of macular pigment is inversely correlated with the incidence of AMD. As previously mentioned, only two macular pigments are uniquely accumulated in the human retina from blood plasma, with lutein identified throughout the retina and zeaxanthin being the predominant component in the central macula [44].

Photoreceptor membranes containing polyunsaturated fatty acids susceptible to photooxidation occur in the retina at high oxygen tension and under chronic light exposure. Ocular exposure to sunlight and UV light directed at the lens and retina of the human eye can lead to the induction of retinal degeneration. In photooxidation reactions, the chromophore in the eye absorbs light and produces free radicals such as singlet oxygen, superoxide, and hydroxyl radical, which cause lipid peroxidation, leading to damage to the integrity of biological membranes, resulting in ocular tissue damage and macular degeneration. In addition, phototoxic reactions can modify histidine, tryptophan, and cysteine, some amino acids of the lens, leading to changes in their physical properties, aggregation, and cataracts [45]. Overproduction of free radicals can cause damage to the RPE [46,47], Bruch’s membrane [48], and choroid, which are layers in the eye involved in the pathophysiology of AMD [49].

Exposure to visible light (400–700 nm), blue light (400–525 nm), and UV radiation from sunlight (220–400 nm) leads to simultaneous photochemical isomerization of retinal chromophores and activation of photoreceptors (e.g., rhodopsin, lipofuscin, melanin) coupled to the chromophores [50]. Photoreceptor cells (rods and cones) shed their outer segments daily to eventually be phagocytosed by retinal pigment epithelial (RPE) cells. During digestion, the RPE releases lipofuscin, which increases with age in healthy eyes. It localizes to the lysosomal bodies of the RPE and may occupy ~20% of the cytoplasmic space by age 80 years, contributing to the pathogenesis of AMD [51].

Macular xanthophylls can accept or donate an electron from the polyene chain and can react with superoxide via electron transfer to generate a radical cation or anion. In addition, there are reports that both lutein and zeaxanthin are better scavengers of the hydroxyl radical than the superoxide anion and that zeaxanthin scavenges the hydroxyl radical more effectively than lutein [52,53]. Furthermore, it has been shown that macular xanthophylls can react with alkyl radicals (R*), lipid peroxyl radicals (ROO*), and alkoxy radicals (RO*) from polyunsaturated fatty acid (PUFA) oxidation and inhibit hydroperoxide (ROOH) formation. Alkyl, peroxyl, and alkoxy radicals are formed in the order of their appearance in the free radical chain reaction. Rodrigues et al. [53] report that both xanthophylls, lutein and zeaxanthin, show a similar ability to scavenge superoxide radicals.

**Figure 4:**
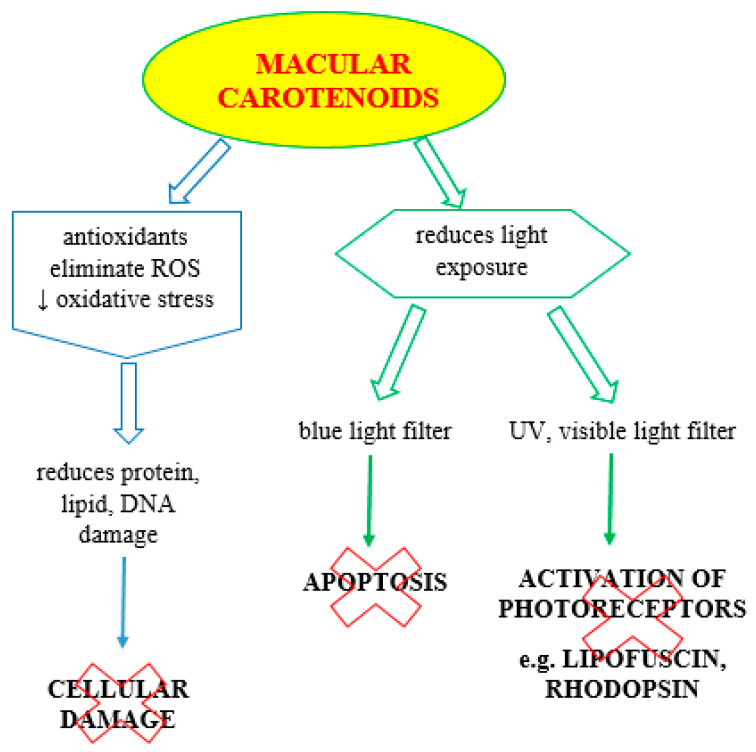
Overview of potential mechanisms of macular pigment action (original).

Lutein and zeaxanthin can filter blue light to prevent the formation of reactive oxygen species, especially singlet oxygen, in the retina and can further reduce oxidation by directly quenching singlet oxygen and related free radicals in the retina [50]. Possible mechanisms have been proposed for how macular xanthophylls exert their protective function. A specific transmembrane orientation of macular xanthophylls has been proposed for quenching reactive oxygen species and protecting the retina from AMD. A study by Subczynski W et al. [54] documented that macular pigment located transversely in the lipid bilayer of the retinal membrane can prevent AMD. This proposed arrangement in the membrane bilayer also protects the retina from peroxidation and photodamage by acting as antioxidant quenchers of RFTs and ROS. The mechanisms responsible for the action of lutein and zeaxanthin include prevention of phototoxic damage by absorbing solar radiation, reduction of oxidative stress by scavenging ROS, and antioxidant, antiangiogenic, and anti-inflammatory properties, as shown in Figure 4.

Furthermore, GSTP1 protein plays an important role in protecting the retina from oxidative damage. GSTP1, as a pi-class glutathione S-transferase (GST) isoform, has been identified as a zeaxanthin- and mesozeaxanthin-binding protein and is localized in the retina. The localization of GSTP1 in the macula as a zeaxanthin-binding protein suggests that GSTP1 also plays an important role in modulating antioxidant levels in the macula [7]. Similarly, human retinal lutein-binding protein (HR-LBP) is a lutein-binding structure. Bhosale et al. [27] documented that HR-LBP may facilitate lutein’s localization to a region of the cell subject to considerable oxidative stress.

Chemical quenching of singlet oxygen by carotenoids has also been shown to lead to oxidation of the quencher. Aldehydes and endoperoxides have been identified as oxidation products of lutein and zeaxanthin, which are involved in various biological functions such as signaling molecules and as photosensitizers in vision [55]. The authors of this study [56] found that lutein oxidation products formed by photooxidation exhibited higher antioxidant and cytotoxic properties than lutein.

Additionally, the chain-breaking activity of macular carotenoids has been shown to be greater than that of vitamin E, a retinal antioxidant. The authors explained that this higher capacity is directly related to the conjugation chain length of the xanthophyll pigment—zeaxanthin, with 11 conjugated double bonds, has a higher singlet oxygen quenching rate constant than lutein, with 10 conjugated double bonds [57,58].

Chemical quenching of singlet oxygen and lipid peroxyl radicals by macular xanthophylls leads to oxidation of the quencher and these molecules are oxidized to their corresponding radical cations. The relationship between antioxidants appears to be very important because these cations must be reduced to regenerate the original carotenoid, allowing them to be reused as antioxidants. Vitamin E can reduce oxidized carotenoids, but this in turn causes tocopherol to remain oxidized. However, oxidized vitamin E can be reduced and regenerated by ascorbic acid. Vitamin C can then be further reduced by copper and zinc. Through this union of oxidation and reduction processes, antioxidants become pro-oxidants that can damage the retina, as found in the AREDS1 clinical trial [59,60,61,62,63].

Results from human studies have shown that the macula, where the pigment concentration is highest, is also the area most resistant to degenerative changes. Studies also confirm that macular pigment concentrations are lower in patients with AMD, and epidemiological results have shown that these are also lower serum concentrations and dietary supply. Therefore, dietary supplementation with lutein and zeaxanthin is now recommended for the prevention of retinal diseases. Modification of a diet rich in the pigment xanthophylls increased their amount in the macula, although no supplementation can replace a well-chosen diet [30,64,65].

The first epidemiological study to demonstrate a direct relationship between lutein intake and AMD risk was reported by Seddon et al. [66]. The AREDS study (2001) [67] is the primary and most important work providing clinical guidance on dietary supplementation. Their premise was to look for opportunities to inhibit the progression of dry AMD and they sought a pathway that would slow the progression to advanced AMD. The randomized AREDS 1 study published in 2001 showed that supplementing the diet with antioxidant vitamins and minerals (AREDS formula) reduces the risk of developing advanced macular degeneration by 25%. Animal studies, epidemiological data, and evaluations of dietary habits in different countries have indicated that the inclusion of lutein + zeaxanthin, and omega-3 unsaturated fatty acids (docosahexaenoic—DHA and eicosapentaenoic—EPA), in the diet reduces the risk of developing advanced AMD. This became the basis for the AREDS 2 study [63]. It was assumed that DHA is an essential structural component of the retina and EPA affects its biological function. Lutein and zeaxanthin in a ratio of 5:1 were supplemented together because lutein absorption is dependent on the presence of zeaxanthin. In addition to visual acuity, progression of AMD to central geographic atrophy or a neovascular form, and serum levels of lutein, zeaxanthin, DHA, and EPA, were determined at clinical follow-up. The primary analysis from the AREDS2 trial suggested that xanthophyll supplementation provided no further benefit regarding the rate of AMD progression, compared with the original AREDS formulation. However, secondary analysis showed that the progression of dry AMD to advanced AMD was 31% lower in the AREDS plus lutein and zeaxanthin groups compared to placebo. This correlated with increased serum lutein and zeaxanthin concentrations of 190–210% in the first to fifth year of the study compared with the placebo group, where they remained unchanged. Results from the AREDS2 study showed a beneficial effect of adding lutein and zeaxanthin to the AREDS1 formulation—at a ratio of 5:1, which reduces the progression of dry AMD to advanced AMD compared to the AREDS1 study (31% versus 25%). The AREDS formulations are shown in Table 2.

Carotenoids are important in AMD because of their physiological functions and location in the retina. Lutein and zeaxanthin are part of the macular pigment. Trace minerals such as zinc and copper may also be involved in retinal antioxidant functions. Omega-3 fatty acids have been shown to have protective effects against macular degeneration, including anti-angiogenic, anti-inflammatory, and antioxidant effects. Omega-3 fatty acids reduce pathological angiogenesis in various cellular and animal models by affecting multiple angiogenic factors, including platelet-derived growth factor (PDGF) and vascular endothelial growth factor (VEGF) [68,69]. Unsaturated fats also facilitate the absorption of lutein and zeaxanthin.

Regular consumption of more foods such as spinach, kale, and cabbage has been shown in many studies to provide significant protection against the onset of late AMD [67,70,71,72].

There are many studies available in the literature that have shown that supplementation with xanthophyll carotenoids can significantly improve their serum levels [63,64,73,74,75,76,77,78] and levels within retinal tissue among AMD patients [64,73,75,76,79,80,81,82,83,84,85,86,87].

There are also studies showing that higher dietary antioxidant intake, including with lutein and zeaxanthin, can significantly reduce early AMD associated with genetic risk variants. Polymorphisms that modify AMD susceptibility have been found in at least 37 genes. One of the most important is a single-nucleotide polymorphism in the complement factor H gene at location 1q26. The presence of the unfavorable Y402H variant increases the risk of developing any form of AMD, and also increases the risk of faster disease progression and poorer response to treatment [88]. The risk of AMD among carriers of the CFH Y402H variant increased up to 11-fold, and up to 15-fold in carriers of the ARMS2 variant [89,90,91,92].

## 5. Conclusions

The possibility of using modern research techniques may provide new evidence for the effective role of lutein and zeaxanthin in the etiology of AMD. Thus far, the treatment options for the dry form of AMD are very limited, and all efforts are limited to inhibiting progression to advanced forms of this degeneration, i.e., geographic atrophy and the exudative form. The AREDS 2 study conducted between 2006 and 2012 developed a formulation of dietary supplements that can reduce the risk of progression to advanced forms at certain stages of the disease. This risk is reduced by around 25% in people with moderate disease in both eyes or moderate disease in one eye and advanced disease in the other eye.

Research continues into other treatments for dry AMD, including methods using nanosecond laser-2RT, orally administered drugs (emixustat), or intravitreal preparations (lampalizumab, sirolismus, pegcetacoplan) [93,94,95,96]. Thus far, the results of using these forms of therapy are not satisfactory. Other forms of treatment, independent or complementary, are also being sought.

The effects of the inhibition of other growth factors, such as angiopoietin (Faricimab) [97], the use of a Port Delivery System (PDS) with ranibizumab [98], or gene therapy [99] are being tested.

It appears that, in aging retinal tissue, inhibition of endogenous antioxidant capacity, marked by a decrease in macular xanthophylls—lutein, zeaxanthin, and mesozeaxanthin—is a major contributor to AMD progression. The use of adjunctive therapy with carotenoid phytochemicals in clinical treatment algorithms for AMD seems to be justified. It has been demonstrated that adjunctive therapy with carotenoid phytochemicals not only provides neuroprotection but may also have a beneficial effect on treatment strategies at any stage of AMD, even advanced AMD.

## Figures and Tables

**Table 1:** Xanthophyll content in different foods. Modified from Kwiatkowska E. [3].

Food Product	Lutein/Zeaxanthin mg/100 g
kale	39.55
spinach	11.93
lettuce	2.63
broccoli	2.44
brussels sprouts	1.59
green peas (canned)	1.35
corn (canned)	0.88
green beans	0.64
carrot	0.35
cabbage	0.31
melon	0.04

**Table 2:** AREDS formulations.

	AREDS1 Dose/Day	AREDS2 Dose/Day
Vitamin C	500 mg	500 mg
Vitamin E	400 IU	400 IU
Beta-carotene	15 mg	-
Zinc	80 mg	25 mg
Copper	2 mg	2 mg
Lutein	-	10 mg
Zeaxanthin	-	2 mg

## Data Availability

Data is contained within the article.
